# ^188^ Re-N-DEDC Lipiodol for Treatment of Hepatocellular Carcinoma (HCC)—A Clinical and Prospective Study to Assess In-Vivo Distribution in Patients and Clinical Feasibility of Therapy

**DOI:** 10.1055/s-0043-1764306

**Published:** 2023-05-01

**Authors:** Naresh Kumar, Priyanka Gupta, Shamim Ahmed Shamim, Viju Chirayil, Suresh Subramanian, Madhava B. Mallia, Chandrasekhar Bal

**Affiliations:** 1 Department of Nuclear Medicine, All India Institute of Medical Sciences (AIIMS), New Delhi, India; 2 Bhabha Atomic Research Centre, Mumbai, Maharashtra, India

**Keywords:** hepatocellular carcinoma (HCC), trans-arterial radionuclide therapy (TART), DEDC (diethydithiocarbamate), radiochemical purity (RCP), radiochemical yield (RCY)

## Abstract

**Objective** The incidence of inoperable hepatocellular carcinoma (HCC) with/without malignant portal vein thrombosis (PVT) is increasing in India for the last decade; thus, Bhabha Atomic Research Centre (BARC), Mumbai, India, developed diethydithiocarbamate (DEDC), a new transarterial radionuclide therapy (TART) agent. ^188^ Re-N-DEDC lipiodol is an emerging radiotherapeutic agent for inoperable HCC treatment due to its simple and onsite labeling procedure, cost-effectiveness, and least radiation-induced side effects. This study aimed to evaluate in-vivo biodistribution and clinical feasibility of ^188^ Re-N-DEDC lipiodol TART in HCC and optimization of labeling procedure to assess post-labeling stability and radiochemical yield of labeled lipiodol with ^188^ Re-N-DEDC complex.

**Materials and Methods** DEDC kits were obtained as gift from BARC, Mumbai. Therapy was given to 31 HCC patients. Post-therapy planar and single-photon emission computed tomography/computed tomography (SPECT/CT) imaging were performed to see tumor uptake and biodistribution. Clinical feasibility and toxicity were decided by Common Terminology Criteria for Adverse Events version 5.0 (CTCAE v 5.0).

**Statistical Analysis** Descriptive statistics was done for data using SPSS v22. Values was expressed as mean ± standard deviation or median with range.

**Results** Post-therapy planar and SPECT/CT imaging showed radiotracer localization in hepatic lesions. Few patients showed lungs uptake due to hepato-pulmonary shunt (lung shunt < 10%). Maximum clearance was observed through urinary tract with very less elimination through hepatobiliary route due to slow rate of leaching of tracer. No patient showed myelosuppression or any other long-term toxicity over median follow-up of 6 months. Mean overall % radiochemical yield of ^188^ Re-N-DEDC lipiodol was 86.04 ± 2.35%. The complex ^188^ Re-N-DEDC was found to be stable at 37°C under sterile condition over a period of 1 hour without any significant change on the % radiochemical purity (90.83 ± 3.24%, 89.78 ± 3.67%, 89.22 ± 3.77% at 0, 0.5, 1 hours, respectively).

**Conclusion** Human biodistribution showed very high retention of radiotracer in hepatic lesions with no long-term toxicity with this therapy. The kit preparation procedure is ideally suited for a busy hospital radio-pharmacy. By this procedure, ^188^ Re-N-DEDC lipiodol can be prepared in high radiochemical yield within a short time (∼45 minutes). Thus, ^188^ Re-N-DEDC lipiodol can be considered for TART in advanced and/or intermediate HCC.

## Introduction

Hepatocellular carcinoma (HCC) is one of the most common cancers, being fifth most frequent malignancy and the third leading cause of cancer death worldwide. 1 A range of 70 to 95% of HCC occurs in cirrhotic patients in Asian population. 2 3 Hepatitis B or C viruses, direct chronic exposure to aflatoxins, chronic alcohol consumption (> 50–70 g/day for prolonged time), and use of estrogen-containing oral contraceptives (> 5 years) are the well-established risk factors for HCC. 4 The highest age standardized incidence as well as mortality rate per 100,000 population is notable in Mongolia in East Asia. Globally, the diverse HCC incidence is due to the variable prevalence of associated etiologies. 5 A recent publication projecting HCC incidences in 30 countries worldwide that predicts the percentage change in age standardized incidence rate over a period of 25 years, from 2005 to 2030. There is a drop in chronic viral hepatitis-related HCC incidences, with an increased nonalcoholic steatohepatitis (NASH)-related HCC incidences by 122% between 2016 and 2030 in United States. 6 7

The prognosis for HCC is extremely poor, and the treatment depends on the disease extent and staging. The Barcelona Clinic Liver Cancer (BCLC) staging system is the most common and frequently used staging in HCC. This includes four components, that is, tumor extension, liver functional reserve (Child-Pugh stage), physical status (Eastern Cooperative Oncology Group [ECOG] performance status), and cancer-related symptoms. In addition to prognostication, the BCLC staging system also recommends appropriate management options as per each stage. The curative treatment (surgery or local ablative procedures) can only be carried out in only 20 to 30% of cases in very early stage (stage 0) and early stage (stage A). 8 Intermediate stage (stage B) and advanced stage (stage C) patients may receive palliative treatments such as radio frequency ablation, transarterial chemoembolization, selective internal radiation therapy (SIRT)/transarterial radioembolization (TARE), or systemic therapy. 9 10 Currently, sorafenib or lenvatinib is the standard first-line systemic therapy for advanced stage HCC patients. 11 12 End-stage patients (stage D) often receive symptomatic treatment or best supportive care. 8

SIRT/TARE is an internal targeted radiation therapeutic technique with the use of various β-emitting radionuclides (such as yttrium-90, holmium-166, iodine-131, and rhenium-188) for the locoregional treatment for intermediate or advanced HCC. 13 ^90^ Y-microsphere is contraindicated in patients with severe abnormal liver function because of its embolic nature, the cost per therapy up to 10 times higher than ^131^ I-lipiodol and Rhenium-188 nitrido-diethyldithiocarbamate ( ^188^ Re-N-DEDC) lipiodol therapy, and significant bone marrow toxicity with ^90^ Y-labeled compounds because of high rates of leaching over time. 14 ^188^ Re-N-DEDC lipiodol is an emerging agent for transarterial radionuclide therapy (TART) in HCC patients. Rhenium-188 has a half-life of 16.9 hours, high beta energy (E _βmax_ = 2.1 MeV) close to ^90^ Y (E _βmax_ = 2.28 MeV) with t _1/2_ = 64.1 hours, and comparable maximum range of 11 mm in tissue. Low gamma energy 155 keV (15% abundance) emission is appropriate for monitoring of the localization of radiopharmaceutical in the target tissue, imaging, and patient-specific dosimetry. 15

The improved 4-hexadecyl-4, 7-diaza 1, 10-decanedithioacetate (AHDD) kits conjugated with ^188^ Re-lipiodol were safely used in HCC patients (BCLC-B and C). 16 Unfortunately, the radiochemical yield (RCY) is limited to under 70 to 80%; therefore, higher activity needs to be added to synthesize desired activity that may lead to high exposure to researcher. 16 17 Due to unpredictable and low RCY, researchers are looking for an alternative to AHDD having similar biodistribution and clinical feasibility but higher RCY with ^188^ Re. DEDC labeled with ^188^ Re is another TART agent that had proven its efficacy for the therapy of unresectable liver cancer. The preclinical trials with ^188^ Re-N-DEDC lipiodol showed retention of activity in liver with no detectable levels of activity in lungs, kidneys, or any other vital organs. 18 The preparation of DEDC kits require addition of stipulated quantity of glacial acetic acid that might lead to low radiochemical purity (RCP) of ^188^ Re-N-DEDC complex and less RCY than expected if any error in quantity of acetic acid. Most of radiopharmacy operations prefer acetic acid free preparation; therefore, recently an improved freeze-dried kit developed by Bhabha Atomic Research Centre (BARC), Mumbai, that uses sodium oxalate buffer which can significantly improve the %RCP of the complex. Thus, the recently developed improved DEDC kit can be used for ^188^ Re-N-DEDC lipiodol labeling to overcome the drawbacks with AHDD kits. 19

This study aimed for the optimization of improved labeling procedure to get maximum RCY, assessment of stability of ^188^ Re-N-DEDC complex at optimal temperature, and final %RCY in lipiodol phase. Also, the study prospectively assessed the human in-vivo biodistribution and feasibility of TART with ^188^ Re-N-DEDC lipiodol from clinical as well as radiation protection point of view.

## Materials and Methods

### Radiochemistry and Standardized Labeling Procedure

Lyophilized freeze-dried kits (containing 2 vials [vial-1 and vial-2]) of DEDC were obtained as gift from BARC, Mumbai, India. The kits were prepared under sterile condition and sterility was checked prior to supply by BARC. The labeling was performed by using freshly eluted ^188^ Re-sodium-perrhenate obtained from commercial ^188^ W/ ^188^ Re-generator (PARS Rhen ^188^ W/ ^188^ Re generator). Vial 1 contains 2 mg N-methyl-S-methyl dithiocarbazate (DTCz), 10 mg sodium ascorbate, 28 mg oxalic acid, 0.8 mg SnCl _2_ .2H _2_ O, while vial 2 contains 100 mg DEDC in freeze dried form. The species DTCz was an efficient nitrido donor (N ^3−^ ), SnCl _2_ as reducing agent and oxalic acid played important role in expanding the coordination sphere of the ^188^ Re-(VII) ion required for improved radiopharmaceutical yield. ^188^ Re-labeled-lipiodol was obtained by dissolving ^188^ Re-N-DEDC complex in lipiodol (lipiodol ultra-fluid, Guerbet, France).

**Fig. 1:**
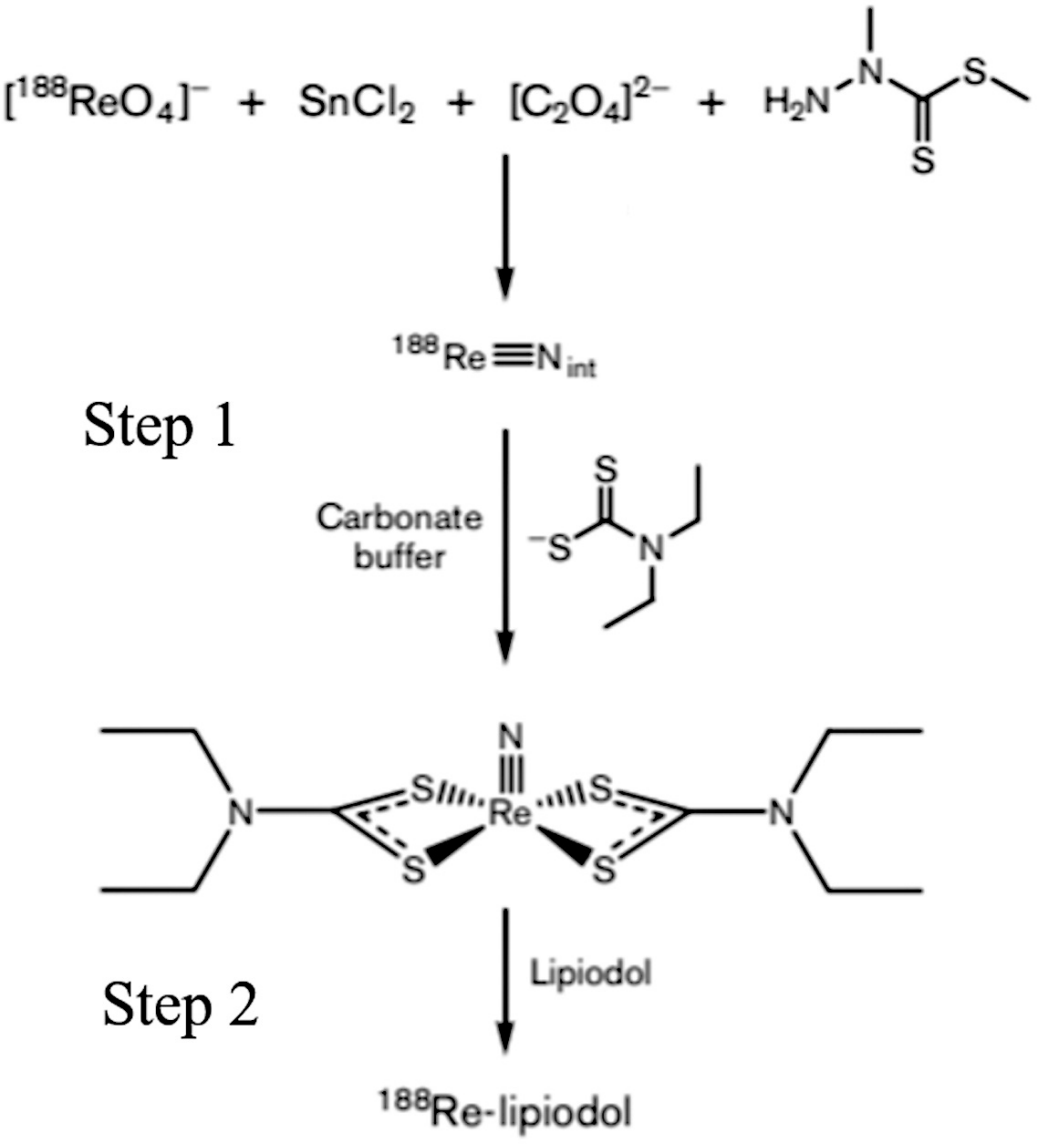
Schematic diagram showing the reactions involving the preparation in Rhenium-188-N diethyldithiocarbamate ( ^188^ Re-N-DEDC) lipiodol. Step 1 involved the formation of ^188^ Re-N-DEDC complex following this step 2 involved addition of lipiodol and obtained final ^188^ Re-N-DEDC lipiodol.

The standard labeling procedure involved the addition of required sodium perrhenate activity (independent of volume) to vial-1 that form intermediate ^188^ Re-nitrido complex ( ^188^ Re ≡ N ^2+^ ). After 5 minutes of incubation, 1 mL reconstituted solvent of vial 2 was added to the vial 1 that led to formation of yellow color precipitate confirming the correct labeling procedure. Further, the intermediate mixture was made to stand for 15 minutes at room temperature and required more incubation at 65°C for 5 minutes in a water bath, thus enabling the formation of ^188^ Re-N-DEDC complex. Lipiodol (up to 4 mL) was added in labeled ^188^ Re-N-DEDC complex. The solution was thoroughly homogenized for approximately 10 minutes with a rotary vortex mixer and then centrifuged at 3,500 rpm for 15 minutes at 4°C. Thus, radiolabeled lipiodol was extracted from bottom of mixture vial using a spinal needle under controlled air conditions by putting an air vent in vial for easier separation of both layers. The reactions involving the formation of ^188^ Re-N-DEDC lipiodol given in Fig. 1 .

After ^188^ Re-N-DEDC complex formation, %RCP of complex was assessed as the percentage ratio of the radioactivity of labeled ^188^ Re-N-DEDC complex over the total activity of free ^188^ Re-sodium perrhenate. %RCP was carried out with thin-layer chromatography (TLC) silica gel (SG) 60 F _254_ aluminum-backed strips obtained from Merck (Darmstadt, Germany) or Whatman paper no. 1 as stationary phase; dichloromethane (Fisher scientific, New Hampshire, United states) as mobile phase and counting was performed by well counter (Biodex Atomlab 950 thyroid uptake system with optional well counter).

About 0.5 µL (upto 3 µCi) of ^188^ Re-N-DEDC complex was applied on two different TLC strips and developed into dichloromethane. After drying with air drier, one strip was cut into two equal halves and other was cut into ten equal halves; the radioactivity of each part was counted using well counter to assess %RCP and retention factor (R _f_ ) of the complex, respectively. The piece with maximum count indicated the retention factor of the complex.



The %RCY was calculated as the percentage ratio of final radioactivity of ^188^ Re-N-DEDC lipiodol obtained after its radiochemical separation from aqueous free perrhenate and total activity added in reaction vial-1 during preparation. The post-labeling stability of ^188^ Re-N-DEDC complex was checked to assess %RCP at various time points 0, 0.5, 1, 2, 3, 6, 12, and 24 hours at 37°C under sterile condition. Also, the %RCY of ^188^ Re-N-DEDC-lipiodol after 24 hours was also performed. The reproducibility of radiolabeling was assessed in 18 tests with DEDC kits.

## Human Biodistribution and Clinical Feasibility

Radiologically and/or histopathologically confirmed intermediate/advanced HCC patients having ECOG performance status of 2 or less and Child-Pugh score A/B were prospectively included in this study. Written informed consent was taken from all the patients. Ethical clearance for the study was obtained from institute ethical clearance committee (IECPG-755/23.12.2021, RT-03/27.01.2022) for ^188^ Re-N-DEDC lipiodol TART. Following the labeling procedure, the therapeutic activity (1.5–5 GBq) was injected through tumor feeding artery depending upon tumor size under fluoroscopic guidance through femoral branch in super-selective manner. 18 Planar and single-photon emission computed tomography (SPECT) imaging was performed on Mediso Any Scan SPECT/CT system (Budapest, Hungary) at 2, 6, 12, 24, 48, and 72 hours in nuclear medicine department to see tumor uptake and biodistribution of ^188^ Re-N-DEDC lipiodol. Planar images were acquired at a scan speed of 150 mm per minute for anterior and posterior whole-body image with matrix size is 256 × 1,024 and zoom factor is 0.88 (2.73 mm/pixel), SPECT was acquired in continuous mode at projection arc of 2.77 degree with time per projection of 17 seconds, matrix size is 128 × 128 and zoom factor is 1.14 (4.25 mm/pixel), and CT was acquired at 2 hours imaging time along with SPECT at exposure is 260, tube current (mA) is 390 and tube voltage (kV) is 120.

Clinical feasibility of therapy was decided on the basis of early or late toxicity evaluations. Clinical and laboratory (investigational) toxicities were graded in accordance with Common Terminology Criteria for Adverse Events version 5.0 (CTCAE v5.0) developed by the National Cancer Institute of the USA.

### Radiation Exposure

The whole-body radiation dose was measured using electronic pocket dosimeter (ALOKA MYDOSE mini, PDM-222-SH, Southern Scientific Ltd., UK).

### Statistical Analysis

Descriptive statistics has been done for data using SPSS v22. Values have been expressed as mean ± standard deviation or median with range. Data is expressed as mean and number and percentage for qualitative and quantitative variables.

## Results

### Radiochemistry and Standard Labeling Procedure

**Fig. 2:**
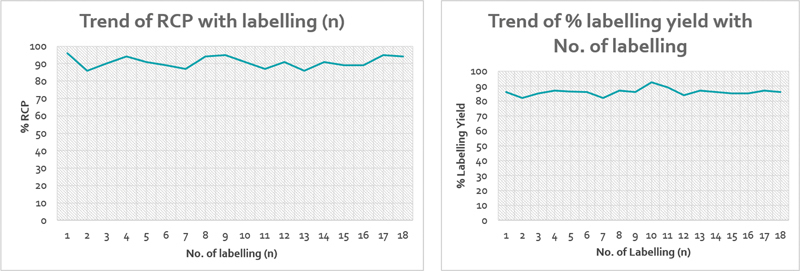
The trend of percentage of radiochemical purity (%RCP) and radiochemical yield (%RCY) with total number of labeling procedures.

Standard labeling procedure using improved DEDC kits containing sodium oxalate buffer took only 45 minutes for synthesis of ^188^ Re-N-DEDC lipiodol that is less than previously described procedure as well as other commercially available kits. 16 19 The radiolabeling parameters such as %RCP, retention factor of complex, and %RCY in lipiodol phase were assessed in 18 tests with DEDC kits. The data showing number of labeling procedures, activity added, %RCP, and %RCY are given in Table 1 . Fig. 2 shows the trend of %RCP and %RCY with the number of labeling procedures.

**Table 1:** Results of all 18 labeling procedures performed for labeling of ^188^ Re-N-DEDC lipiodol

S.no. (labeling)	Activity added (mCi)	Activity before lipiodol addition (mCi)	Free activity (mCi)	Labeled lipiodol (mCi)	% RCY in lipiodol phase	%RCP of complex
1	67	65	9	56	86	96
2	48	47	8.5	38.5	82	86
3	70	69.5	10	59	85	90
4	40	40	5	35	87	94
5	100	99	13.6	85.4	86.3	91
6	52	51	7	44	86	89
7	37	36	6.5	29.5	82	87
8	48	47	6	41	87	94
9	32	31	4.4	26.6	86	95
10	44	44	3.3	40.7	92.5	91
11	26	25	3	22	89	87
12	42	41	6.6	34.4	84	91
13	26	25	3.3	21.7	87	86
14	45	45	6	39	86	91
15	24	23	3.5	19.5	85	89
16	40	39	6	33	85	89
17	38	38	5	33	87	95
18	25	24	3.4	20.6	86	94

Abbreviations: ^188^ Re-N-DEDC, Rhenium-188-N diethyldithiocarbamate; %RCP, percentage radiochemical purity; %RCY, percentage radiochemical yield.

**Fig. 3:**
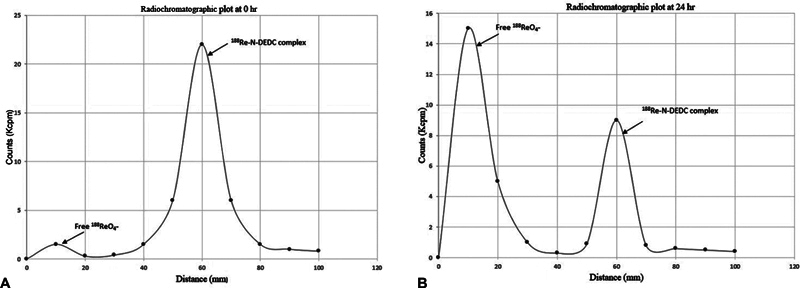
Radiochromatograph plot to determine percentage of radiochemical purity (%RCP) of Rhenium-188-N diethyldithiocarbamate ( ^188^ Re-N-DEDC) complex ( **A** ) immediately after labeling having % RCP > 90% with R _f_ = 0.6 ( **B** ) at 24 hours after labeling having %RCP approximately 30%.

On observation, it was noticed that the free sodium perrhenate was spotted at origin, while the labeled complex was present at 0.6 to 0.7 on silica gel or Whatman strip. The radiochromatograph plot to assess %RCP at 0 and 24 hr is indicated in Fig. 3 . The mean %RCP immediately after labeling of complex was 90.83 ± 3.24. The resulted R _f_ was obtained as 0.6 on TLC radiochromatograph ( Fig. 3A ).

**Fig. 4:**
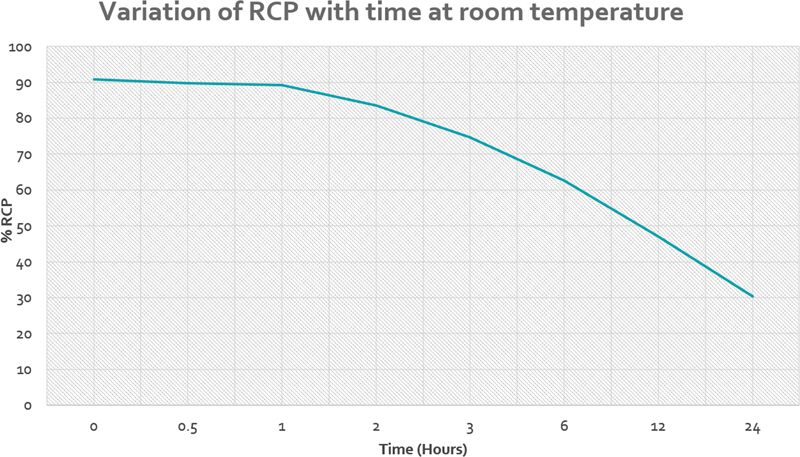
The variation in percentage of radiochemical purity (%RCP) with time (hours) up to 24 hours indicated that the Rhenium-188-N diethyldithiocarbamate complex was almost stable till 1 hour with very less detectable change on % RCP.

The stability of complex was decided by %RCP at room temperature that was 89.78 ± 3.67, 89.22 ± 3.77, 83.57 ± 3.2, 74.71 ± 5.18, 62.57 ± 3.20, 47 ± 4.43, and 30.28 ± 4.54 at 0.5, 1, 2, 3, 6, 12, and 24 hours, respectively. The variation of %RCP ^188^ Re-N-DEDC complex with time is given in Table 2 and Fig. 4 . The mean overall %RCY of ^188^ Re-N-DEDC lipiodol was 86.04 ± 2.35% ( Table 1 ). In other eight samples, lipiodol (up to 4 mL) was added after 24 hours incubation of ^188^ Re-N-DEDC complex (8 samples) at room temperature. Mean %RCY in lipiodol phase with ^188^ Re-N-DEDC complex after 24 hours was 70.57 ± 3.69%. Also, it was observed that there were no clear boundaries between labeled lipiodol and free perrhenate phase after 24 hours, thus the separation of labeled lipiodol with ^188^ Re-N-DEDC complex was not as clear as immediately after labeling of ^188^ Re-N-DEDC complex.

**Table 2:** Variation in %RCP of ^188^ Re-N-DEDC complex over time

Time after labeling (hours)	% RCP (mean ± SD)
0	90.81 ± 3.24
0.5	89.78 ± 3.67
1	89.22 ± 3.77
2	83.57 ± 3.2
3	74.71 ± 5.18
6	62.57 ± 3.20
12	47 ± 4.43
24	30.28 ± 4.54

Abbreviations: ^188^ Re-N-DEDC, Rhenium-188-N diethyldithiocarbamate; % RCP, percentage radiochemical purity; SD, standard deviation.

### Human Biodistribution and Feasibility of Study

**Fig. 5:**
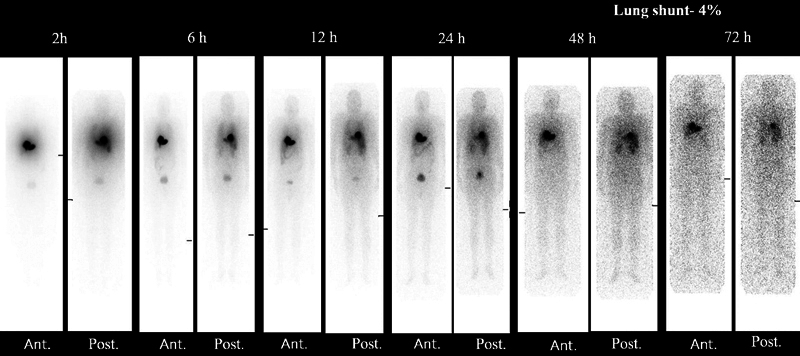
A 52-year-old male with incidentally detected hepatitis B virus-related hepatocellular carcinoma with portal vein thrombosis underwent Rhenium-188-N diethyldithiocarbamate lipiodol transarterial radionuclide therapy; the post-therapy whole-body planar imaging up to 72 hours showing localized retention of lipiodol in liver seg. II/III lesion, faint visualization of kidneys in 2 to 6 hours with maximum visualization in 24 to 48 hours, minimal small intestine tracer uptake over 12 to 24 hours, and high bladder uptake due urinary excretion.

**Fig. 6:**
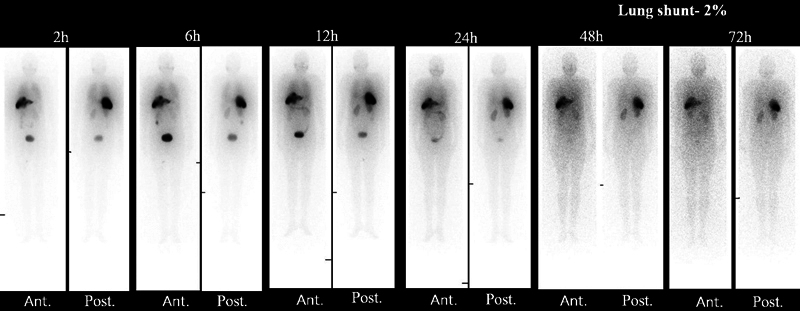
A 52-year-old male with a known case of hepatitis-C virus and non-alcoholic steatohepatitis -related hepatocellular carcinoma with portal vein thrombosis underwent Rhenium-188-N diethyldithiocarbamate lipiodol transarterial radionuclide therapy; the post-therapy whole-body imaging showing localized tracer uptake up to 72 hours in seg. VI lesion, minimal uptake in salivary gland due to free perrhenate, faint visualization of kidneys in 2 to 6 hours with maximum visualization in 24 to 48 hours, minimal gut uptake over time (12–24 hours), and high bladder uptake due to urinary excretion.

**Fig. 7:**
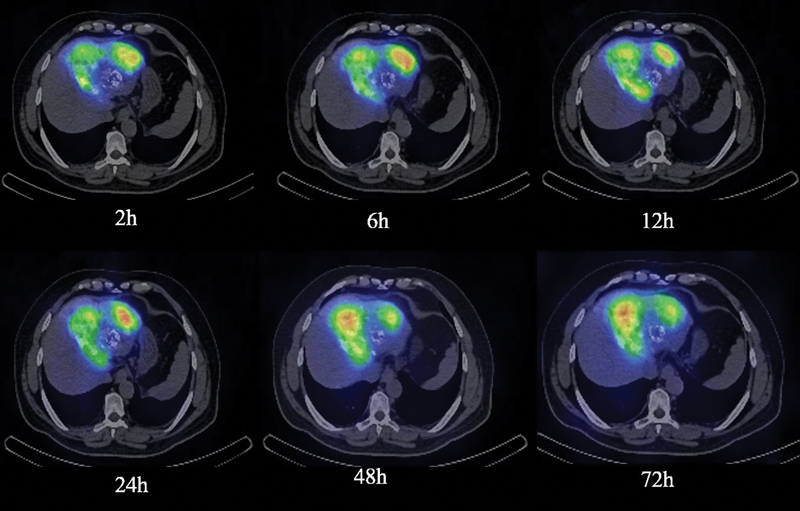
Co-registered single-photon emission computed tomography/computed tomography images of liver up to 72 hours of above-mentioned patient in Fig. 5 after transarterial administration of Rhenium-188-N diethyldithiocarbamate lipiodol, showing high retention of activity in seg II/III lesion.

**Fig. 8:**
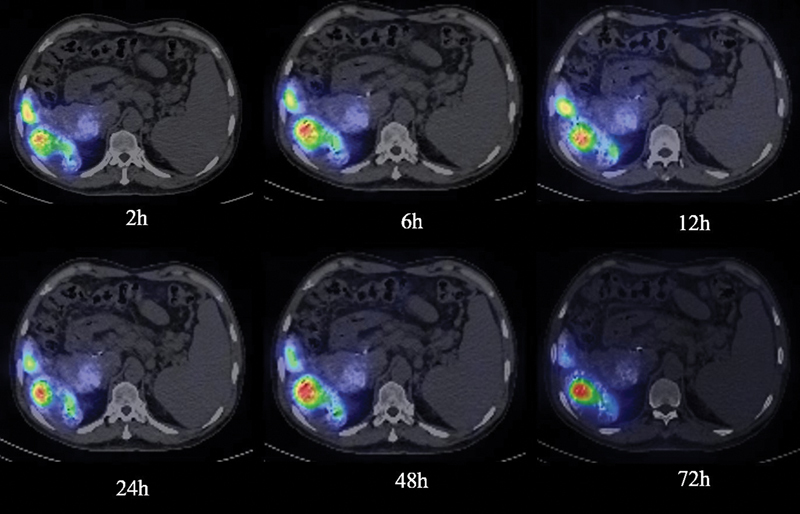
Co-registered single-photon emission computed tomography/computed tomography images of liver up to 72 hours of above-mentioned patient in Fig. 6 after transarterial administration of Rhenium-188-N diethyldithiocarbamate, showing high retention of activity in seg VI lesion.

Thirty-one (27 male, 4 female) patients with mean age of 55.9 ± 9.78 years of intermediate/advanced staged HCC patients have been treated with ^188^ Re-N-DEDC lipiodol TART. Overall mean injected activity of ^188^ Re-N-DEDC lipiodol was 2.9 ± 0.9 GBq (78.4 ± 24.2 mCi). Their biodistribution showed localized retention of lipiodol inside the lesion up to 72 hours on planar ( Figs. 5 and 6 ) and SPECT/CT ( Figs. 7 and 8 ) imaging with only six patients (~19%) showing mild lung uptake due to hepatopulmonary shunt. All six patients had hepatopulmonary shunt less than 10%; thus, dose reduction was not required in any patient. Faint visualization of kidneys was observed at 2 to 6 hours whole-body imaging with maximum uptake in 24 to 48 hours due to urinary route of elimination. Digestive tract such as small intestine showed increased ^188^ Re-N-DEDC lipiodol uptake over time (12–24 hrs) due to their lipophilic characteristic and slow elimination through hepatobiliary tract. High bladder uptake was due to urinary excretion as the main route. Mild uptake of free perrhenate was seen in thyroid and salivary glands of only five patients (~16%).

All 31 patients were available for toxicity evaluation, and the median follow-up time was of 6 months (range: 3–12 months). None of the patient had myelosuppression or any other long-term toxicity. Post-therapy clinical toxicities (nausea, vomiting, fever, and abdominal pain) were observed in most of the patients for 2 to 3 days and were treated symptomatically. One patient showed grade 3 liver toxicity and progressive worsening of liver function test, 20 patients showed grade 1 derangements in liver enzymes, and six patients showed grade 2 derangements in liver enzymes and later on toxicity was managed conservatively in hospital lasting for 3 days. Hematological toxicities were seen in four patients (≤ grade 2) in 2 patients and grade 3 in two patients. The patient with grade 3 hematological toxicity had either low hemoglobin or low platelet counts at baseline as well as follow-up and required red blood cell (RBC) transfusion or fresh frozen plasma infusion post-therapy, respectively. None of the patient had any pulmonary toxicity that were quantitatively seen by comparing baseline and follow-up pulmonary function test. Patient characteristics, biodistribution, and toxicity evaluation data are given in Table 3 .

**Table 3:** Patient characteristics, biodistribution, and toxicity evaluation of patients after ^188^ Re-N-DEDC lipiodol administration

Patient characteristics	
Gender ( *n* = 31)	Male = 27 Female = 4
Age	Mean = 55.9 ± 9.78 years
Injected activity	Mean = 2.9 ± 0.9 GBq (78.4 ± 24.2 mCi)
**Biodistribution**	
Mild lung uptake	*n* = 6 (~19%)
Mild thyroid uptake	*n* = 5 (~16%)
**Toxicity evaluation (CTCAE v5.0)**	
Grade I derangement in AST/ALT (> ULN–3.0 x ULN)	*n* = 20 (64.5%)
Grade II derangement in AST/ALT (> 3.0–5.0 x ULN)	*n* = 6 (19.3%)
Grade III liver toxicity	*n* = 1
Hematological toxicity Grade II Grade III	*n* = 2 *n* = 2

Abbreviations: AST/ALT, aspartate aminotransferase/alanine aminotransferase; CTCAE v5.0, Common Terminology Criteria for Adverse Events version 5.0; ^188^ Re-N-DEDC, Rhenium-188-N diethyldithiocarbamate; ULN, upper limit of normal range.

The post-therapy dosimetry was performed in all patients using Monte Carlo methods to estimate radiation absorbed dose to tumor, normal non-neoplastic liver and lungs, and subsequently the radiological, biochemical, and clinical response were assessed at least up to 6 months but are outside the scope of this article. Interestingly, the absorbed dose to normal liver parenchyma and lungs did not exceed 20 and 4 Gy, respectively, in any patient, which is well below the maximum tolerated liver dose of 30 and 12 Gy for lungs.

During the entire synthesis procedure, that is, from elution to post-injection imaging, mean whole-body radiation, dose received by radiation personnel measured by using the pocket dosimeter, was 18 ± 4.73 µSv. The mean whole-body radiation dose during synthesis, quality control (QC), interventional procedure, and imaging was 9.8 ± 2.06, 1.44 ± 0.51, 3.44 ± 0.98, and 3.27 ± 1.18 µSv, respectively.

## Discussion

Low standard reduction potential (E ^0^ ) of ^188^ Re is the major problem in reducing the metal center and also strongly limits the possibility to obtain high yield of ^188^ Re-labeled radiopharmaceuticals by using standard radiopharmaceutical labeling approaches. 20 Boschi et al in 2003 suggested coordination sphere expansion from tetrahedral into square pyramidal or octahedral without changing the starting metal oxidation state by using weakly acidic oxalate ions (C _2_ O _4_^2-^ ) to improve the RCP of the complex and radiochemical yield of final radiopharmaceutical. 21 Boschi et al in 2004 reported that the maximum volume up to 0.9 mL ^188^ Re activity can be added to the kit vial 1 to obtain the maximum radiochemical yield. For the same, the more concentrated activity was required to prepare sufficient patient dose of ^188^ Re-N-DEDC lipiodol, which was laborious and time-consuming process. 18 Thus, this study used improved DEDC kits containing sodium oxalate buffer (0.5 M) instead of glacial acetic acid that shortens the labeling time to maximum 45 minutes and required only 5 minutes incubation for formation of intermediate ^188^ ReN ^2+^ . Also, the perrhenate activity could be added independent of their volume. The mean %RCY of lipiodol and %RCP of ^188^ Re-N-DEDC complex obtained were 86.04 ± 2.35% and 90.83 ± 3.24%, respectively. The short labeling period resulted to least whole-body effective dose to the radiation worker, which was 9.8 ± 2.06 µSv during synthesis.

Radhakrishnan et al in 2022 compared the overall % radiochemical yield and %RCP of freeze-dried kits of AHDD, Super-Six sulphur, and DEDC labeled in lipiodol with ^188^ Re. The mean overall % RCY and % RCP using modified DEDC kits were 87.17% ± 2.7% and 95.43% ± 2.3%, respectively. Also, the modified DEDC kits had advantage of less preparation time and any volume of perrhenate activity that can be added to the kit vial. 22 This study also showed comparable results with improved DEDC kits having mean % RCY and % RCP of 86.04 ± 2.35% and 90.83 ± 3.24%, respectively.

Boschi et al in 2004 reported an inevitable degradation of RCP over time due to radiolysis during preparation of ^188^ Re-N-DEDC lipiodol. The initial % RCP immediately after labeling was over 98% and declined to 50.2% over 24 hours. 18 In our study, we assessed the post-labeling stability of ^188^ Re-N-DEDC complex up to 24 hours. The complex was almost stable till 1 hour with very less impact on RCP in 1 hour. The %RCP of complex was 90.83 ± 3.24% immediately after complex formation and then declined to 30.28 ± 4.54% in 24 hours ( Table 2 ). In practice, the lipiodol wase added to ^188^ Re-N-DEDC complex within 1 hour of formation.

Boschi et al in 2004 in a preliminary clinical study in 12 patients with ^188^ Re-N-DEDC lipiodol reported excellent tumor uptake in the liver, without significant activity in the gut and kidneys, and no lung activity in 1 to 4 hours whole-body gamma imaging. At 20 hours whole-body imaging, the activity was retained in liver lesions with a minimal increase in colon and some uptake in the spleen and, on occasion, the bone marrow. All patients were evaluated to see any clinical and/or laboratory toxicities. Only one patient with portal vein thrombosis had grade 4 myelosuppression due to high dose of ^188^ Re-loaded lipiodol (6 Gbq). The patients with any hematological toxicity were recovered after platelet transfusion and granulocyte colony-stimulating factor therapy. Also, the patients with ^188^ Re-loaded lipiodol activities less than 6 GBq in three treatments over a period of 12 months manifested no deterioration in hematological or biochemical parameters. Finally, they decided to prescribe an upper limit of 5GBq of ^188^ Re-lipiodol activity in further clinical trials for intrahepatic arterial ^188^ Re-lipiodol therapy of unresectable HCC. 18 Our phase II clinical trial also showed almost similar results in human biodistribution study and clinical feasibility of intra-arterial therapy with ^188^ Re-N-DEDC lipiodol. The mean overall injected activity of ^188^ Re-N-DEDC lipiodol was less than 5 Gbq. Human biodistribution showed excellent ^188^ ReN-DEDC lipiodol retention inside the lesion up to 72 hours on planar and SPECT/CT imaging with mild lung uptake due to hepato-pulmonary shunt (< 10%). Both kidneys were faintly visualized at 2 to 6 hours whole-body imaging with maximum uptake in 24 to 48 hours. The increased gut activity (small intestine) over time (12–24 hours) was due to their lipophilic characteristic and slow elimination through hepatobiliary tract. High bladder uptake was due to urinary excretion as the main route ( Figs. 5 and 6 ). Also, toxicity was evaluated in all patients in median follow-up time up to 6 months. None of the patient had myelosuppression or any other long-term toxicity. Most of the patients had mild post-therapy clinical toxicities (nausea, vomiting, fever, abdominal pain) and were treated symptomatically. Patients with liver enzymes derangements were managed conservatively in hospital lasting for 3 days. Patients with hematological toxicities were treated accordingly with RBC transfusion or fresh frozen plasma infusion post-therapy whichever required ( Table 3 ).

Thakral et al in 2018 estimated the whole-body radiation exposure to radiopharmacist during synthesis of ^188^ Re-labeled radiopharmaceuticals. The radiation exposure to radiopharmacist during labeling of ^188^ Re-HDD lipiodol in mean time of 95 minutes was 52 µSv, 23 while our study estimated the mean whole body radiation dose of 9.8 ± 2.06 µSv during synthesis of ^188^ Re-N-DEDC lipiodol in a mean time of only 45 minutes using standard labeling method.

## Conclusion

This study reports a simple and user-friendly kit by addition of calculated amount of oxalic acid and disodium oxalate in improved DEDC kits which eliminated the need of glacial acetic acid for the preparation of ^188^ Re-N-DEDC lipiodol, a clinically established radiopharmaceutical for the treatment of inoperable HCC. This modification has led to a significant simplification of the procedure and required less synthesis time of 45 minutes and less whole-body radiation dose of 10 µSv during in-house synthesis of this therapeutic agent. Thus, the whole-body effective dose received by personnel involved was well within recommended safety levels of occupational dose limits of Atomic Energy Regulatory Board (AERB), India, that is, 20 mSv/year (averaged over 5 years); so, it can be concluded that manual synthesis of ^188^ Re-N-DEDC lipiodol is safe in radiation protection point of view. The biodistribution study showed excellent retention of activity in liver lesions and toxicity evaluation reported no long-term toxicity or myelosuppression to any patient. Thus, ^188^ Re-lipiodol, prepared with indigenous prepared DEDC kits, is clinically safe and effective therapy for the treatment of intermediate/advanced HCC when given through super selective tumor feeding artery.
